# Simultaneously Selective Detection of Trace Lead and Cadmium Ions by Bi-Modified Delaminated Ti_3_C_2_T_x_/GCE Sensor: Optimization, Performance and Mechanism Insights

**DOI:** 10.3390/ma18122828

**Published:** 2025-06-16

**Authors:** Ruhua Peng, Kai Tao, Baixiong Liu, Jiayu Chen, Yunhang Zhang, Yuxiang Tan, Fuqiang Zuo, Caihua Song, Xingyu He

**Affiliations:** 1 School of Materials Science and Engineering, Jiangxi University of Science and Technology, Ganzhou 341000, China; 15207080615@163.com (R.P.); 15868100589@163.com (K.T.); liu_micro@126.com (B.L.); 13879375814@163.com (J.C.); 19532332665@163.com (Y.Z.); 19179998791@163.com (Y.T.); 2 Sanchuan Wisdom Technology Co., Ltd., Yingtan 335000, China; a19532332665@outlook.com (F.Z.); tk15868100589@126.com (C.S.)

**Keywords:** square wave anodic stripping voltammetry, Bi nanoparticles, sensitivity, actual water

## Abstract

Lead (Pb) and cadmium (Cd) ions have serious negative impacts on human health and the ecological environment due to toxicity, persistence and nonbiodegradability. Among various trace Pb and Cd ions detection technologies, electrochemical analysis is considered as one of the most promising methods. The deposition of Bi nanoparticles on delaminated Ti_3_C_2_T_x_ (DL-Ti_3_C_2_T_x_) develops a sensor with good conductivity and performance. Square wave anodic stripping voltammetry (SWASV) technology was applied to simultaneously deposit Bi on DL-Ti_3_C_2_T_x_/GCE and achieve the rapid detection of Pb and Cd ions. The Bi nanoparticles effectively improved the sensitivity of Bi/DL-Ti_3_C_2_T_x_/GCE sensors to detect Pb and Cd ions. The preparation conditions of the Bi/DL-Ti_3_C_2_T_x_/GCE were optimized, including DL-Ti_3_C_2_T_x_ droplet amount, solution pH, Bi^3+^ concentration, deposition time and deposition potential, to improve the detection ability. The Bi/DL-Ti_3_C_2_T_x_/GCE sensor has detection limits of 1.73 and 1.06 μg/L for Pb and Cd ions, respectively (S/N > 3). This electrochemical sensor is easy, sensitive and selective to apply in actual water samples for trace Pb and Cd ions detection.

## 1. Introduction

With the development of global industrialization, the impact of human activities is constantly expanding, such as mining and metal ore smelting, waste gas emissions and various types of waste caused by human household waste [1,2]. Heavy metal pollution accounts for a significant proportion of severe environmental pollution problems [3]. Heavy metals (HMs) refer to naturally occurring metal elements with atomic densities higher than 4.5 g/cm^3^ and similar metal-like elements including lead, cadmium, mercury, copper, zinc and iron [4]. Due to the high stability, wide distribution, difficulty in biodegradation and high toxicity of heavy metals, they are prone to causing irreversible damage to the surrounding environment [5]. They can also interfere with normal physiological functions of the human body through biological accumulation in the food chain, posing persistent and toxicological effects on human health [6,7]. Among them, lead, cadmium, chromium, mercury and metals like arsenic are the most toxic [8]. Cadmium and lead ions are the most typical in aquatic environments [9]. Excessive exposure to cadmium ions can easily lead to hypertension, cardiovascular and cerebrovascular diseases, damage to bone calcium and renal dysfunction [8,10]. Lead poisoning may damage the blood and nervous system, affecting children’s intelligence and growth [11]. Therefore, it is necessary and important to develop sensitive and rapid detection methods for Pb and Cd ions.

The traditional methods for detecting heavy metal ions include Inductively Coupled Plasma Optical Emission Spectrometry (ICP-OES) [12], Inductively Coupled Plasma Mass Spectrometry (ICP-MS) [13], High Performance Liquid Chromatography (HPLC) [14], Auger Electron Spectroscopy (AES) [15], Atomic Absorption Spectrometry (AAS) [16] and X-ray Fluorescence Spectrometry (XRF) [17]. Although these methods can achieve specificity and sensitivity in detecting most metal ions, they have drawbacks such as expensive instrument equipment, large volume, the need for professional detection personnel and the inability to perform real-time on-site detection, which limits their application in heavy metal detection [18,19]. Electrochemical detection methods (especially anodic stripping voltammetry) are considered as one of the most promising methods for trace heavy metal ions detection due to their advantages of excellent sensitivity, high efficiency and economy. In addition, electrochemical detection methods combine the portability of instruments and can achieve real-time in situ detection, making them highly developed in environmental science [20,21].

Due to their equivalent or better electrochemical performance and low toxicity, Bismuth membrane electrodes have become promising alternatives to mercury droplets and membrane electrodes for heavy metal ions detection [22,23]. Bismuth electrodes also have the advantages of high stability, insensitivity to dissolved oxygen, resistance to reactions with oxygen or water and resistance to interference from dissolved oxygen during testing. In addition, the excellent electrochemical sensor performance of bismuth electrodes can form binary or multicomponent alloys with other heavy metals, rather than competing with heavy metals for surface active sites [23]. Therefore, bismuth metal electrodes with high sensitivity, low toxicity, easy electrode surface renewal and good reproducibility have significant advantages in trace heavy metal ions determination [24].

In recent years, various nanomaterials (including carbon nanotubes, graphene oxide, etc.) have been combined with bismuth to further improve the reproducibility and sensitivity of bismuth electrodes [24,25]. For example, Huang et al. [25] constructed a new, cost-effective portable BiNDs/P-GE sensor that achieves excellent synchronous Pb^2+^ and Cd^2+^ detection performance and exhibits low detection limits and high sensitivity, repeatability, stability and anti-interference capabilities. Wen et al. [26] introduced Bi^3+^ into the MXA-CuO/CC sensing system using the synergistic adsorption of oxygen vacancy and Bi^3+^ on heavy metal ions to construct a novel MXene aerogel-CuO/carbon cloth electrochemical sensor that can simultaneously detect Cd^2+^ and Pb^2+^. The detection limits were 0.3 μg/L and 0.2 μg/L in the linear ranges of 4 to 800 μg/L and 4 to 1200 μg/L, respectively.

Among various modification materials, two-dimensional (2D) materials have received widespread attention due to their wide applications. Among them, Ti_3_C_2_T_x_ MXene is a kind of novel metal carbon/nitride with a two-dimensional layered structure [27,28]. Compared with traditional two-dimensional materials, Ti_3_C_2_T_x_ MXene has the advantages of good biocompatibility, high conductivity, strong stability, high hydrophilicity and abundant active sites (i.e., functional groups such as -F, -OH, -O) [29,30,31,32]. Therefore, Ti_3_C_2_T_x_ MXene has good application prospects in fields such as electrochemical sensors, supercapacitors and catalysis [33,34]. In addition, it has been shown that material resistivity increases with the increase of MXene thickness, while the single-layer Ti_3_C_2_T_x_ MXene sheet shows better electrical conductivity [35], and the modified Ti_3_C_2_T_x_ MXene sheet has a high density of functional groups, which can improve the material loading capacity and accelerate the rate of analyte acquisition [36,37]. Therefore, this research selected delaminated Ti_3_C_2_T_x_ (DL-Ti_3_C_2_T_x_) as the carrier for designing electrochemical sensors.

In this study, SWASV was used to simultaneously deposit Bi on DL-Ti_3_C_2_T_x_/GCE and achieve rapid Pb and Cd ions detection. The preparation conditions of Bi/DL-Ti_3_C_2_T_x_/GCE were optimized, including DL-Ti_3_C_2_T_x_ droplet amount, detection solution pH, Bi^3+^ concentration, deposition time and deposition potential, to improve the detection ability for Pb and Cd ions in solution.

### Reagents

The reagents are presented in the Supplementary Materials Text S1.

## 2. Materials and Methods

### 2.1. Preparation of Ti_3_C_2_T_x_ MXene Nanosheets

Previously, the precursor MAX (Ti_3_AlC_2_) was treated with in situ hydrofluoric acid etching (HCl + LiF → HF + LiCl) for chemical etching and delamination of the Al layer [38,39]. Firstly, LiF (3.2 g) was added in a 100 mL high-temperature resistant PTFE beaker. Then, HCl (9 M, 50 mL) was added and stirred for 30 min. Ti_3_AlC_2_ powder (2 g) was slowly added and continuously stirred at 45 °C for 48 h. Then, the suspension was washed with deionized water. The black liquid was sonicated for 3 h and then centrifuged to obtain a black solid. The black solid was dried in a vacuum freeze-drying machine to finally obtain DL-Ti_3_C_2_T_x_ nanosheets.

### 2.2. Electrode Preparation

#### 2.2.1. GCE Pretreatment and Preparation

Exposed glassy carbon electrode (GCE) was polished with a suede polishing cloth using alumina powder. The electrode was oriented perpendicular to the polishing cloth and was cycled in an “8”-shaped or circular trajectory with 10 clockwise and 10 counterclockwise turns until the electrode surface became smooth and flat. After polishing, the electrodes were cleaned with deionized water, nitric acid (1:1), ethanol and deionized water by ultrasonic cleaning for 30 s, and then dried under an infrared lamp. The test conditions are presented in Text S2. The potential difference between the reduction and oxidation peaks of the CV curve was 60~80 mV, indicating that the electrode was polished clean and could be used for subsequent experiments. The cyclic voltammetry is shown in Figure S1.

#### 2.2.2. Preparation of DL-Ti_3_C_2_T_x_/GCE

On the surface of the GCE, DL-Ti_3_C_2_T_x_/GCE was prepared by a drop coating method. The prepared DL-Ti_3_C_2_T_x_ nanosheets were dissolved in ultrapure water to sonicate (power, 500 W) for 2 h, then a uniformly dispersed DL-Ti_3_C_2_T_x_ suspension (1 mg/mL) was obtained. A volume of 8 μL of Ti_3_C_2_T_x_ suspension droplets with a pipette was accurately transferred onto the pre-treated GCE surface. The electrode surface was dried under infrared light to prepare the DL-Ti_3_C_2_T_x_/GCE.

#### 2.2.3. Preparation of Bi/DL-Ti_3_C_2_T_x_/GCE

There were two methods used for preparing the bismuth film electrodes: a pre-plating bismuth film method and an in situ plating bismuth film method. The deposition potential of bismuth was −1.2 V, and the deposition time was 270 s on the electrochemical workstation. Under stirring conditions, bismuth was enriched and deposited on the electrode surface to prepare the Bi/DL-Ti_3_C_2_T_x_/GCE. The quantitative determination of heavy metals was carried out by a co-plating bismuth film method, which involved adding the tested ions to the electrolyte containing Bi^3+^ solution. During the detection process, the tested heavy metal ions (Cd, Pb) and Bi^3+^ precipitated simultaneously on the DL-Ti_3_C_2_T_x_/GCE to prepare the DL-Ti_3_C_2_T_x_/GCE, and the other conditions were the same as above.

### 2.3. Electrochemical Characterization of Bi/DL-Ti_3_C_2_T_x_/GCE

The CV curve was obtained using scanning cyclic voltammetry. In the EIS experiment, under the condition of 0.22 V, the scanning frequency was set between 0.01 and 1,000,000 Hz to obtain the EIS graph.

### 2.4. Bi/DL-Ti_3_C_2_T_x_/GCE Detection Experiment for Heavy Metal Ions

The electrolyte was a 0.2 M acetate buffer solution containing Bi^3+^ (pH = 4.5) and Cd and Pb ions in the solution were detected via SWASV, which involved two steps: enrichment and dissolution. The enrichment process parameters were as follows: the sedimentation potential was −1.2 V and the sedimentation time was 270 s. The relevant parameters of the dissolution process are as follows: scanning potential: −1.0~−0.2 V, amplitude: 0.050 V, amplification potential: 0.004 V and settling time: 2 s. Finally, electrochemical cleaning was performed on the electrode at a constant voltage of 0.2 V. Before preparation and testing, high-purity N_2_ was used to remove O_2_ from the electrolyte.

## 3. Results and Discussion

### 3.1. Material Morphology

**Figure 1:**
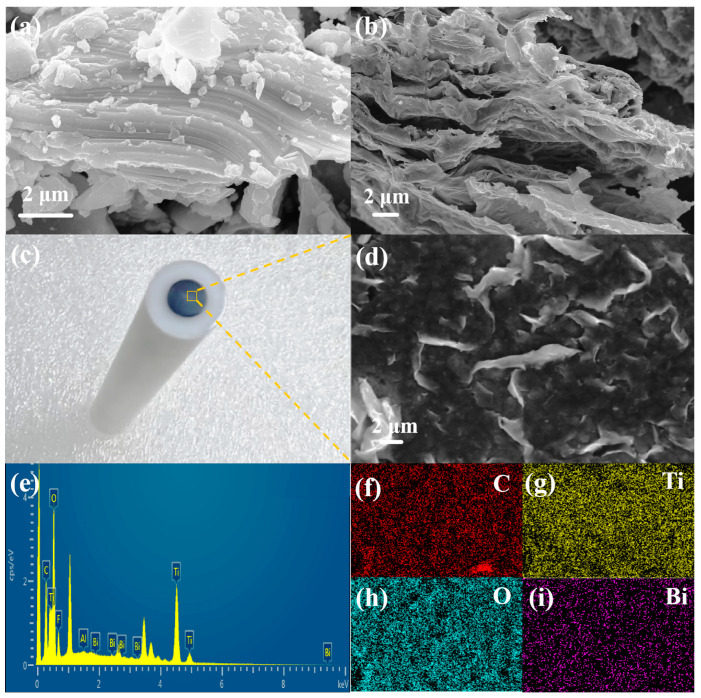
(**a**) SEM images of precursor Ti_3_AlC_2_ MAX and (**b**) DL-Ti_3_C_2_T_x_; (**c**) Bi/DL-Ti_3_C_2_T_x_/GCE; (**d**) SEM images of Bi/DL-Ti_3_C_2_T_x_; (**e**) EDS results of Bi/DL-Ti_3_C_2_T_x_; (**f**–**i**) Bi/DL-Ti_3_C_2_T_x_ corresponding to C, Ti, O, Bi element mapping.

The microstructure and morphology of precursor Ti_3_AlC_2_ MAX and DL-Ti_3_C_2_T_x_ were characterized using TEM and SEM. The precursors Ti_3_AlC_2_ (Figure 1a) and DL-Ti_3_C_2_T_x_ (Figure 1b) both have 2D layered structures. The precursor MAX in Figure 1a is a layered and dense blocklike structure. In Figure 1b, DL-Ti_3_C_2_T_x_ exhibits a clear layered structure, which was attributed to the destruction of Ti Al bonds between the layers after HF selective etching, resulting in the peeling of each layer and the formation of loosely arranged multilayer Ti_3_C_2_T_x_. Figure S2 shows the elemental distribution images of the precursor Ti_3_AlC_2_ MAX and DL-Ti_3_C_2_T_x_. Compared with the precursor, the Al content in the etched MXene is significantly reduced. The distribution of C, O, F and Ti elements is clearly displayed, which further confirms the effective etching of the Al layer by HF [40]. Figure 1c shows the prepared Bi/DL-Ti_3_C_2_T_x_/GCE sensor, and the composite material on the electrode surface is characterized by SEM (Figure 1d). Layered stacking folds of DL-Ti_3_C_2_T_x_ flakes were observed, and the elemental distribution (Bi, O, C and Ti) of Bi/DL-Ti_3_C_2_T_x_ was identified; Bi nanoparticles are uniformly distributed on the surface of DL-Ti_3_C_2_T_x_, confirming the successful synthesis of the Bi/DL-Ti_3_C_2_T_x_ (Figure 1e–i) [41,42].

The synthesized composite materials were characterized using TEM. The TEM images of the precursor Ti_3_AlC_2_ MAX phase are presented in Figure S3a,b. A block structure with a size of approximately 3 nm is observed, the lattice spacing is about 0.96 nm. On the other hand, the DL-Ti_3_C_2_T_x_ sample (Figure S3c) shows an almost transparent layered structure, indicating that ultrasonic treatment is beneficial for the layering and peeling of DL-Ti_3_C_2_T_x_ thin films, with a layer spacing of 1.40 nm (Figure S3d). Layered stacking folds were observed in the TEM image of Bi/DL-Ti_3_C_2_T_x_ composite material in Figure S3e, which may be due to the stacking of DL-Ti_3_C_2_T_x_ droplets during the drying process on the GCE surface. This is consistent with the SEM image analysis results, with a measured lattice spacing of 1.56 nm (Figure S3f). Combined with SEM characterization analysis, HF effectively etched the Al layer and successfully peeled it off to prepare DL-Ti_3_C_2_T_x_. The modification of Bi further increased the specific surface area of the composite material, which is beneficial for effectively enhancing the detection performance of the electrode sensor.

Figure S4 presents the XRD spectra of Ti_3_C_2_T_x_ MXene and Bi/DL-Ti_3_C_2_T_x_ composite materials. It can be observed that the main characteristic peaks of the MAX phase (JCPDS NO.52-0875) at 9.5°, 19.1°, 39.0° and 41.8° correspond to the (002), (004), (104) and (105) planes, respectively [43]. Compared with the MAX phase, many diffraction peaks of the MXene material had weakened or disappeared after etching; the disappearance of the characteristic peak at 39.0° indicates that the Al had been removed by etching. After etching, the (002) peak shifted from 9.5° to 6.3°, clearly moving towards a smaller angle, indicating that the interlayer spacing was increased after etching and intercalation treatment. According to the Bragg equation, the interlayer spacing enlarged from 0.96 to 1.40 nm. The reason was that Al was removed from the MAX phase. Compared with the (002) peak of Ti_3_C_2_T_x_ MXene, the spectral peak of Bi/DL-Ti_3_C_2_T_x_ shifted to 5.67° (1.56 nm), indicating that surface modification of Bi on DL-Ti_3_C_2_T_x_ increased the interfacial spacing, which was consistent with the analysis of TEM spectra.

**Figure 2:**
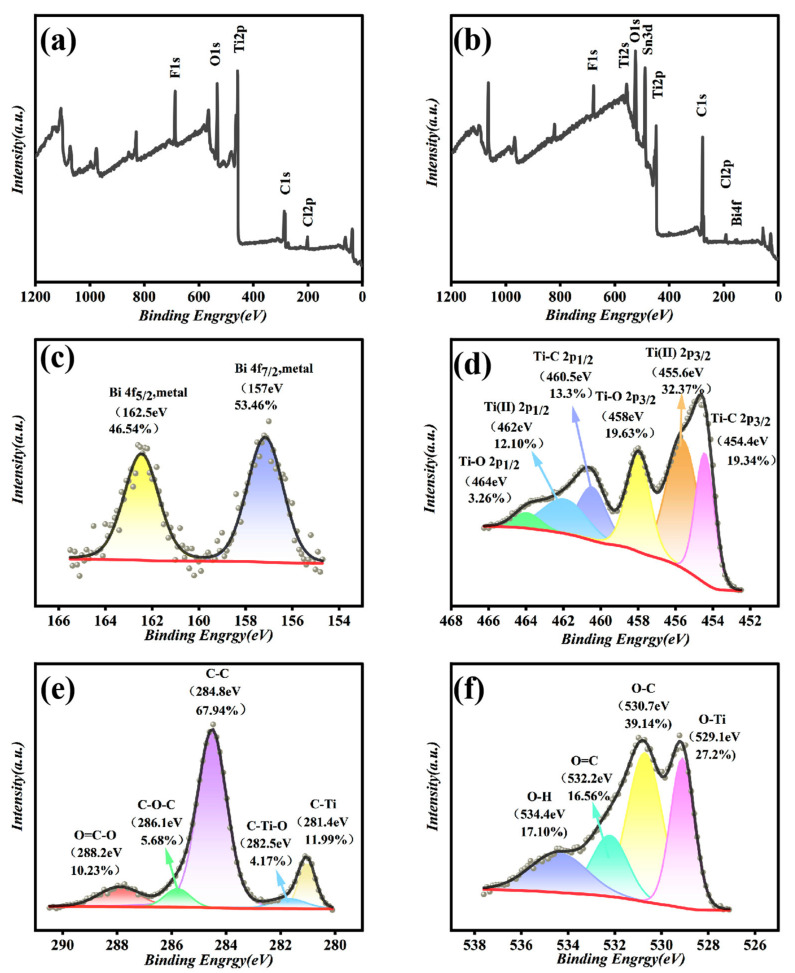
(**a**) XPS image of DL-Ti_3_C_2_T_x_; XPS images of Bi/DL-Ti_3_C_2_T_x_ (**b**) and XPS spectra of Bi 4f (**c**), Ti 2p (**d**), C 1s (**e**) and O 2s (**f**) in Bi/DL-Ti_3_C_2_T_x_.

The chemical bonding state of Bi/DL-Ti_3_C_2_T_x_ composite materials were analyzed using XPS. The XPS spectrum of Bi/DL-Ti_3_C_2_T_x_ composite material is shown in Figure 2. Compared with the precursor MAX phase (Figure S5), no Al element was detected in the composite material, indicating that Al was completely etched and peeled off. In addition, elemental Bi appeared in the Bi/DL-Ti_3_C_2_T_x_ composite material, and an Sn 3d peak was detected, which was derived from conductive glass doped with tin fluoride. Combining the spectra of Bi 4f (Figure 2c), two peaks at 162.5 and 157.0 eV correspond to the 4f_5/2_ and 4f_7/2_ orbitals of metal Bi, respectively, further confirming that Bi^3+^ was completely reduced to metallic Bi. As shown in Figure 2d–f, the oxygen-containing functional groups of Bi/DL-Ti_3_C_2_T_x_ are abundant, indicating that Bi nanoparticles do not occupy many oxygen-containing functional groups, which is conducive to the enrichment of trace heavy metals [44].

### 3.2. Electrochemical Characterization

The electrochemical performances of the GCE, DL-Ti_3_C_2_T_x_/GCE and Bi/DL-Ti_3_C_2_T_x_/GCE were characterized and compared using CV and EIS. The electrolyte was a mixture of 0.1 M KCl and 5 mM [Fe(CN)_6_]^3−/4−^ solution, as shown in Figure S6. The CV curve of bare GCE shows a pair of typical [Fe(CN)_6_]^3−/4−^ redox characteristic peaks (Figure S1). Compared with the GCE, the DL-Ti_3_C_2_T_x_/GCE and Bi/DL-Ti_3_C_2_T_x_/GCE sensors show no obvious redox reaction characteristic peaks, and the surface electrode is relatively stable. The CV curve area of the DL-Ti_3_C_2_T_x_/GCE is larger, and the addition of DL-Ti_3_C_2_T_x_ on the surface can effectively improve the conductivity of the electrode (Figure S6a). The CV curve area of the two electrodes is larger, and the addition of DL-Ti_3_C_2_T_x_ on the surface can enhance the conductivity of the electrode (Figure S6a). In the EIS plot, the semicircular diameter in the high-frequency region corresponds to the electron transfer impedance (Ret) of each electrode. The order of Ret values for each electrode is Bi/DL-Ti_3_C_2_T_x_/GCE > GCE > DL-Ti_3_C_2_T_x_/GCE, which is consistent with the CV curve. Therefore, the surface modification of DL-Ti_3_C_2_T_x_ on the electrode can effectively promote electron transfer on the electrode surface, which is beneficial for improving the sensitivity of the sensor.

### 3.3. Experimental Exploration

The feasibility of SWASV was applied to investigate the Bi/DL-Ti_3_C_2_T_x_/GCE sensors for Cd and Pb detection. The obtained dissolution voltammetry curves are presented in Figure S7a,d; Figure S7a,b show that when Bi/DL-Ti_3_C_2_T_x_/GCE sensors were used for detecting Cd and Pb separately, their peak voltages were −0.78 V and −0.52 V, respectively. However, when both metal ions were detected simultaneously (Figure S7c), the peak voltage positions shifted to the right to a certain extent, reaching −0.76 V (Cd) and −0.51 V (Pb), respectively. This may be due to the competition between the deposition of Cd(II) and Pb(II) metal ions and the formation of intermetallic compounds, which interfere with the detection of heavy metal ions throughout the dissolution process.

Three sensing electrodes (GCE, DL-Ti_3_C_2_T_x_/GCE, Bi/DL-Ti_3_C_2_T_x_/GCE) were applied for simultaneous detection of Cd(II) and Pb(II) (Figure S7d). It can be seen that the three electrodes can complete detection of Pb and Cd ions simultaneously, but the peak current of the Bi/DL-Ti_3_C_2_T_x_/GCE sensor is the highest, because DL-Ti_3_C_2_T_x_, as the skeleton of Bi nanoparticles grows, provides more space for Bi load while improving the conductivity of the electrode, and Bi can form similarly to the “amalgam effect” with heavy metals [45], so that it can combine more Cd and Pb, greatly improving the sensitivity of the electrode; thus, the prepared Bi/DL-Ti_3_C_2_T_x_/GCE sensor has a high detection ability to simultaneously determine the presence of Cd and Pb ions.

### 3.4. Optimization Experiment of Bi/DL-Ti_3_C_2_T_x_/GCE Sensor Conditions for Cd and Pb Ions Detection

It is necessary to explore the experimental conditions for electrode preparation to achieve the optimal detection performance of Bi/DL-Ti_3_C_2_T_x_/GCE sensors for Cd and Pb ions. The main influencing conditions were considered in this experiment, including the amount of DL-Ti_3_C_2_T_x_ coating, the pH value of detection electrolyte ABS, the concentration ratio of Bi, the deposition time t, the deposition potential E and the influence of concentration.

To achieve the optimal detection effect of SWASV, the DL-Ti_3_C_2_T_x_ drop coating amount was optimized, as shown in Figure S8a,b. Electrodes with different DL-Ti_3_C_2_T_x_ drop coating amounts were prepared, and their effects on the peak current of Cd and Pb ions dissolution were investigated. At a deposition potential of 1.1 V and a deposition time of 270 s, a single heavy metal ion with a concentration of 100 μg/L was measured. DL-Ti_3_C_2_T_x_ was prepared with drop coating amounts of 2, 4, 6, 8, 10, 12, 14, 16, 18 and 20 µL of electrodes. It is obvious that Cd and Pb ions in the range of 2–8 µL drop coating amounts gradually increase with the increase in drop coating amount, and the dissolution peak current also gradually increases (Figure S8a,b). More drop coating material can provide more active sites. When the dripping amount is 8 μL, the peak current for the dissolution of Cd and Pb ions is the highest. The peak current of dissolution decreases as the drop coating amount exceeds 8 µL. Therefore, the optimal DL-Ti_3_C_2_T_x_ drop coating amount was selected as 8 µL.

The pH of the electrolyte buffer system (0.10 mol/L ABS) is an important factor affecting the current response. Figure S8c,d show that the peak current value of Cd dissolution is higher in ABS buffer solution with a pH range of 5.5, while the Pb peak current value dissolution is highest in ABS solution with a pH of 4.5. This may be because a lower pH can lead to excessive H^+^ in solution, which competes with the measured heavy metal ions. During deposition, it is easier for the glassy carbon electrode to undergo hydrogen evolution reaction and produce bubbles, which is not conducive to enrichment of target ions to be measured and affects the final dissolution peak current value. When the pH increases to near neutral, the hydroxide ion concentration increases, and the hydrolysis reaction of tested heavy metal ions intensifies, thereby affecting heavy metal ions enrichment and leading to a weakened dissolution signal [46]. Therefore, considering all factors, an ABS buffer system with pH = 4.5 was selected for subsequent experiments.

The concentration of bismuth ions is another influencing factor on current response, and electrolytes containing different concentrations of Bi(III) were prepared for the determination of Cd and Pb (Figure S9a,b). The peak current values of Cd and Pb dissolution both gradually increase with the increasing Bi^3+^ concentration. When the concentration is 300 μg/L, the highest dissolution peak current value is achieved. The peak current value gradually decreases with further increasing the concentration of Bi(III). This may be attributed to the low bismuth ions concentration in ABS electrolytic buffer solution that is not conducive to the bismuth oxidation reaction, making it difficult to reduce the deposition activation energy of the tested heavy metal ions. However, an increase in the relative bismuth ions concentration may cause the modified layer to thicken, increasing the interfacial electron transfer resistance [47]. Therefore, the concentration of Bi(III) used for subsequent experiments was 300 μg/L.

Different deposition times result in different amounts of ions deposited on the electrode surface, leading to different dissolution signals. With the increasing deposition time, the amount of Cd and Pb participating on the electrode surface gradually increases (Figure S9c,d). Therefore, the peak current value of dissolution detection of the tested heavy metal ions also increases. However, the deposition time is long; it will cause the material layer on the glassy carbon electrode to become thicker, which is not conducive to the desorption of the tested ions. Moreover, when the amount of Cd and Pb deposited on the sensing electrode surface is in dynamic equilibrium, it is difficult to enrich with more Cd and Pb. Increasing the deposition time will reduce the oxidation peak current [48]. Therefore, the optimal sedimentation time was taken as 270 s.

For Cd and Pb detection, the dissolution peak current values showed an overall trend of first increasing and then decreasing (Figure S9e,f). For Cd, the dissolution peak current increases with the increasing absolute value as the deposition potential ranges from −0.9 V to −1.1 V. The dissolution peak current does not change significantly within the range of −1.1 to −1.2 V, and the response intensity is relatively high. The dissolution peak current decreases in the range of deposition voltage from −1.2 to −1.4 V. For Pb detection, the deposition potential increases with the increasing deposition potential within the range of −0.9 to −1.2 V, and the response intensity is highest at −1.2 V. The reduction potential of the tested heavy metal ion is close to the reduction potential of hydrogen when the deposition potential is less than −1.2 V, causing hydrogen ions to be electrolyzed and produce bubbles. When the deposition potential is greater than −1.2 V, the large deposition potential results in an insufficient degree of deposition, which prevents lead ions from being fully enriched on the glassy carbon electrode in a short period of time [49,50]. Therefore, after comprehensive consideration, for subsequent experiments, −1.2 V was chosen as the optimal deposition potential.

### 3.5. Bi/DL-Ti_3_C_2_T_x_/GCE Sensor for Quantitative Cd and Pb Ions Detection

**Figure 3:**
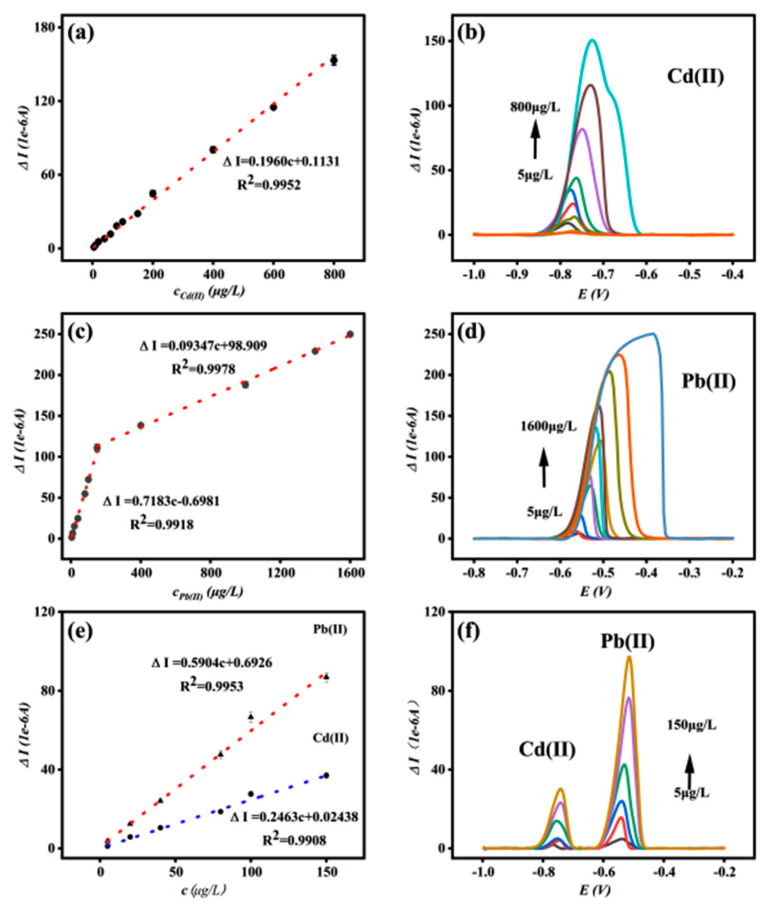
Standard curve graph for Cd(II) detection (**a**) and peak response graph for Cd(II) detection (**b**); standard curve for Pb(II) detection (**c**) and peak response graph for Pb(II) detection (**d**); standard curve for simultaneous Cd(II) and Pb(II) detection (**e**) and peak response graph for simultaneous Cd(II) and Pb(II) detection (**f**) (electrolyte, 0.2 M ABS buffer; pH = 4.5; c[Bi (III)]: 300 μg/L; sedimentation time: 270 s; sedimentation potential, −1.20 V).

Under optimal optimization conditions, SWASV was used to detect Cd and Pb ions using the Bi/DL-Ti_3_C_2_T_x_/GCE sensor prepared above as the working electrode. Figure 3a,b show the standard curve and detection peak response current graph for Cd(II). When the Cd ion concentration range is 5–800 μg/L, the linear fitting equation is ∆I = 0.1960c + 0.1130, R^2^ = 0.9952, and the detection limit (LOD) is 1.73 ppb (S/N = 3). The standard curve and detection peak response current graph of Pb(II) (Figure 3c,d) show that when the Pb ion concentration ranges from 5 to 150 μg/L, the linear fitting equation is ∆I = 0.7183c − 0.6981, R^2^ = 0.9918, and the detection limit (LOD) is 1.06 ppb (S/N = 3). When the concentration of Pb ranges from 150 to 1600 μg/L, the linear fitting equation is: ∆I = 0.09347c + 98.909, R^2^ = 0.9978.

Due to the excellent detection results of the Bi/DL-Ti_3_C_2_T_x_/GCE sensor for both Cd and Pb ions in the above experiment, the ability of the Bi/DL-Ti_3_C_2_T_x_/GCE sensor to simultaneously detect Cd and Pb ions was further explored. Figure 3e,f show the standard curves of both Cd(II) and Pb(II) at different concentrations, as well as the relationship between the peak current values of heavy metal ion detection and concentration. Both Cd(II) and Pb(II) are detected at low concentrations (5–150 μg/L); the linear fitting equation for Cd(II) is ∆I =0.2463c + 0.02438, R^2^ = 0.9908, and the detection limit (LOD) is 1.38 μg/L (S/N = 3); meanwhile, the linear fitting equation for Pb(II) is ∆I = 0.5904c + 0.6926, R^2^ = 0.990, and the detection limit (LOD) is 1.29 μg/L (S/N = 3). The detection limits of both Cd(II) and Pb(II) were not significantly different from those detected separately, indicating that Bi/DL-Ti_3_C_2_T_x_/GCE has good simultaneous detection performance for both Cd(II) and Pb(II) in water. In all the above equations, “c” represents the concentration of metal ions (μg/L), and “∆I” represents the magnitude of the peak response current (1 × 10^−6^ A).

In summary, preparation of the Bi/DL-Ti_3_C_2_T_x_/GCE sensor was simple, and the simultaneous preparation of the Bi/DL-Ti_3_C_2_T_x_/GCE sensor and rapid Cd and Pb ions detection were achieved. As shown in Table 1, compared with many other sensors, the Bi/DL-Ti_3_C_2_T_x_/GCE sensor has obvious advantages of a low detection limit and a wide detection range. In addition, the Bi/DL-Ti_3_C_2_T_x_/GCE sensor, with its low production cost, low pollution and good detection performance, is a promising candidate for Cd and Pb ions detection.

### 3.6. Stability and Repeatability Experiments

**Figure 4:**
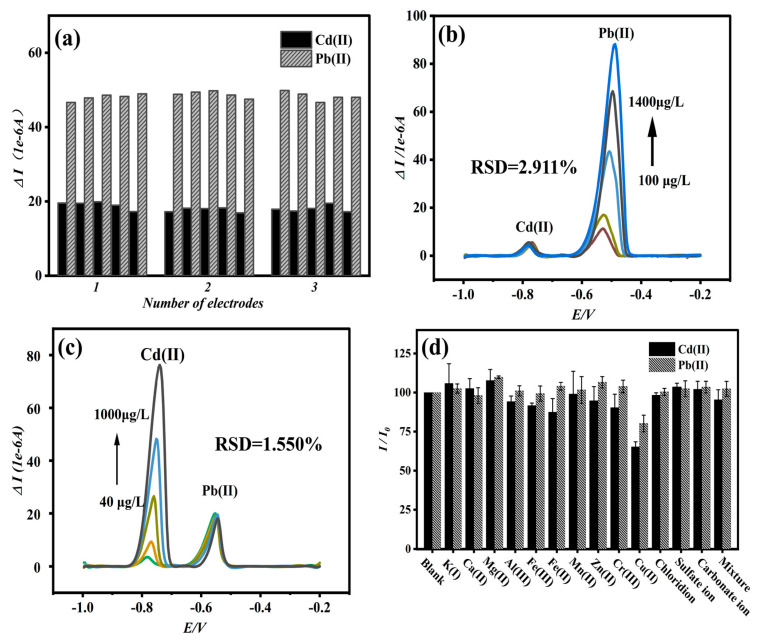
Continuous simultaneous detection of Cd and Pb ions by three electrodes (**a**); the SWASV signal of 100 μg/L Pb with increasing concentration of Cd from 5 to 1000 μg/L (**b**); the SWASV signal of 100 μg/L Cd with increasing concentration of Pb from 10 to 1400 μg/L (**c**); the influence of interference ions on simultaneous detection of Cd and Pb ions (**d**) (electrolyte: 0.2 M ABS buffer; pH = 4.5; c[Bi(III)]: 300 μg/L; c[Pb(II)]: 100 μg/L; c[Cd(II)]: 100 μg/L; interference ions concentration 1000 μg/L; deposition time: 270 s; deposition potential: −1.20 V).

Using the same Bi/DL-Ti_3_C_2_T_x_/GCE sensor as the working electrode, 100 μg/L of Cd(II) and Pb(II) was measured in ABS solution with pH = 4.5. As shown in Figure S10, the dissolution current curves obtained from repeated tests (five times) showed good overlap, and the relative standard deviations (RSDs) of Cd(II) and Pb(II) were calculated to be 5.52% and 1.85%, respectively, proving the stability of the sensor. Three Bi/DL-Ti_3_C_2_T_x_/GCE sensors were prepared, and each electrode was subjected to five repeated tests of Cd(II) and Pb(II) (Figure 4a). The average dissolution current measured by the three electrodes was taken, and the RSDs of Cd(II) and Pb(II) were calculated to be 5.12% and 1.04%, respectively, indicating that the sensing electrode has good repeatability and stability.

### 3.7. Anti-Interference Experiment

To explore the specific detection of Cd and Pb ions by Bi/DL-Ti_3_C_2_T_x_/GCE sensors, anti-interference experiments were conducted. This research conducted simultaneous detection on 100 μg/L Pb and Cd ions by gradually increasing one another’s concentrations. As shown in Figure 4b,c, the peak current values of the controlled concentrations of Pb or Cd ions do not change significantly with increases in one another’s ion concentrations. In other words, the peak current values of Pb and Cd ions with the same concentrations did not change significantly, with RSDs of 2.911% and 1.550%, respectively, while the corresponding peak current values of Cd and Pb ions with the increasing concentrations increased gradually, indicating that the prepared Bi/DL-Ti_3_C_2_T_x_/GCE had excellent stability and selectivity.

### 3.8. Interference Experiments

In real environmental water samples, there may be many interfering ions competing with the tested ions, resulting in a significant deviation between the peak current of the tested ion and the actual situation, thereby affecting the accuracy of the experiment. To mimic the actual environment of water samples (tap water), the anti-interference performance of Bi/DL-Ti_3_C_2_T_x_/GCE sensors against heavy metal ions was tested by manually adding interfering ions. The concentrations of Pb^2+^ and Cd^2+^ ions used in the experiment were 100 μg/L, and the interfering ions were taken from common ions in real water, such as K^+^, Ca^2+^, Mg^2+^, Al^3+^, Fe^3+^, Mn^2+^, Zn^2+^, Cr^3+^, Cu^2+^, Cl^−^, SO_4_^2−^, CO_3_^2−^ and HCO_3_^−^; the concentrations are 1000 μg/L, which is 10 times higher than that of the heavy metal ions tested, making the final interference ion experiment more convincing. From Figure 4d, in addition to Cu^2+^, the interference ions show no obvious interference on the simultaneous detection of Pb^2+^ and Cd^2+^. The Cu(II) may form insoluble alloys with Pb(II) and Cd(II) in the solution, which is not conducive to the detection of Cd(II) and Pb(II) [41,42]. Most coexisting ions had no obvious impact on the peak current of the heavy metal ions measured, indicating that the Bi/DL-Ti_3_C_2_T_x_/GCE sensor demonstrates selectivity in detecting Cd(II) and Pb(II) and is not easily affected by other interfering ions.

### 3.9. Actual Water Sample Testing

To evaluate the applicability of the prepared Bi/DL-Ti_3_C_2_T_x_/GCE sensors, tests were conducted on Cd and Pb ions in actual water samples. The real tap water, Bajiaotang Lake water and Gongjiang River water, were used to prepare a pH = 4.5 acetic acid sodium acetate solution. Pretreatment of sample water samples was carried out as follows: The actual water samples were filtered with a 0.22 μm microporous filter membrane, and then the solution of actual water sample vs. Hac–NaAc solution = 1:9 (*V*:*V*) was prepared to be detected, adopting the standard addition method, with added Cd(II) and Pb(II) standard solution samples to detect three groups of different concentrations (*n* = 3); the concentration and recovery rate of the detection sample were calculated using the standard curve. Each sample was tested in parallel three times. Using the Bi/DL-Ti_3_C_2_T_x_/GCE as the working electrode, water samples were detected for concentrations of Cd and Pb ions using SWASV. Cd and Pb ions with different concentrations were further tested using the standard addition method. The detection results are shown in Table 2. Three sets of tests (*n* = 3) were conducted on Cd and Pb ions, with recovery rates ranges of 98.74~105.33% and 97.86~105.04%, respectively. The detection values basically agreed with the actual amount of Cd and Pb ions. The experimental results showed that the Bi/DL-Ti_3_C_2_T_x_/GCE sensor had high accuracy in actual water sample detection and had the potential to detect Cd and Pb ions pollution in actual water bodies.

### 3.10. Mechanism Analysis

**Figure 5:**
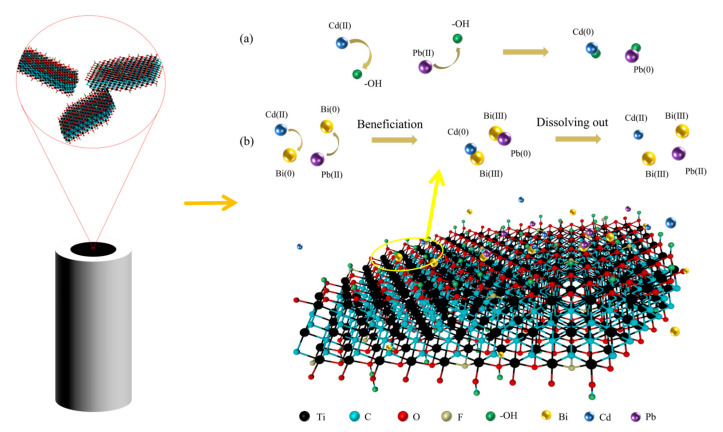
Analysis of Cd and Pb ions detection principle by Bi/DL-Ti_3_C_2_T_x_/GCE sensor: (**a**) Interaction between lead and cadmium ions and the surface functional groups (-OH) of MXene; (**b**) Interaction between lead and cadmium ions and bismuth ions.

The Bi/DL-Ti_3_C_2_T_x_/GCE sensor has excellent Pb and Cd ions detection performance, which may be due to (1) the preparation of the 2D DL-Ti_3_C_2_T_x_ nanomaterial, ultrasonic treatment, and electrodeposition of Bi nanomaterial, which is not only beneficial to the synthesis of fewer layers of DL-Ti_3_C_2_T_x_ but also the layer spacing is greatly increased, so that the prepared composite material has more active sites for metal ions. (2) As shown in Figure 5, the numerous oxygen-containing functional groups on DL-Ti_3_C_2_T_x_ increase the hydrophilicity of the electrode surface. In addition, Cd and Pb ions can form M-O bonds with the functional groups on DL-Ti_3_C_2_T_x_, which is conducive to improving the detection performance of Bi/DL-Ti_3_C_2_T_x_/GCE sensors. (3) The introduction of Bi^3+^ enables uniform Bi nanomaterials to be deposited on the surface of the electrode. Bi nanomaterials not only have the advantages of biocompatibility and a wide negative potential window but also can be combined with some Cd/Pb ions to form alloys, enhancing the adsorption of Cd/Pb ions on the electrode.

## 4. Conclusions

This article explored the preparation of a simple and sensitive Bi/DL-Ti_3_C_2_T_x_/GCE sensor for simultaneous detection of trace amounts of Cd and Pb ions. The detection range of the Bi/DL-Ti_3_C_2_T_x_/GCE is 5–800 and 5–1600 μg/L, with detection limits of 1.73 and 1.06 μg/L for Cd and Pb ions, respectively. In addition, the sensing electrode exhibits good anti-interference performance, excellent stability and reproducibility. In actual water sample experiments, the recovery results calculated using the standard addition method were consistent with the detection results using the standard method. Therefore, the prepared Bi/DL-Ti_3_C_2_T_x_/GCE sensor could achieve sensitive determination of Cd and Pb ions in tap water, reaching its allowable limit. The prepared Bi/DL-Ti_3_C_2_T_x_/GCE sensor is expected to be promoted for water quality inspection in the future.

## Figures and Tables

**Table 1:** Comparison of analytical performance of Cd and Pb ions by different modified electrodes.

Sensor	Detection Technique	Analyte	Linear Range (µg/L)	LOD (µg/L)	Ref.
Bi/GCE	Electrochemical (SWASV)	Cd(II)	5–150	3.2	[51]
		Pb(II)	5–150	1.9	
BiFE/NanoSiO_2_/GCE	Electrochemical (SWASV)	Cd(II)	2–150	0.6	[52]
		Pb(II)	2–150	0.2	
BiOCl/MWCNT/GCE	Electrochemical (SWASV)	Cd(II)	5–50	1.2	[53]
		Pb(II)	5–50	0.57	
BiNPs @CoFe_2_O_4_@GCE	Electrochemical (SWASV)	Cd(II)	9–67.4	0.92	[54]
		Pb(II)	12.4–124	1.51	
GCE/MXene/Nafion	Electrochemical (CV)	Pb(II)	50–2072	10	[34]
Nb_4_C_3_T_x_/GCE	Electrochemical (SWASV)	Pb(II)	5.2–103.6	2.49	[33]
Alk-Ti_3_C_2_/GCE	Electrochemical (SWASV)	Cd(II)	11.2–112	11	[55]
		Pb(II)	20–115	8.50	
Bi/DL-Ti_3_C_2_T_x_/GCE	Electrochemical (SWASV)	Cd(II)	5–800	1.73	This work
		Pb(II)	5–1600	1.06	

GCE, glassy carbon electrode; Alk, alkalization.

**Table 2:** Detection results in actual water.

Sample	Added (μg/L)	Cd(II)				Pb(II)			
		Found (μg/L)	Recovery (%)	RSD (%)	ICP-MS (μg/L)	Found (μg/L)	Recovery (%)	RSD (%)	ICP-MS (μg/L)
Tap water	10	10.24	102.4	3.03	10.01	10.18	101.80	1.37	10.03
	50	50.78	101.56	1.01	50.13	48.93	97.86	1.45	49.60
	100	104.47	104.47	1.40	100.25	104.63	104.63	1.01	100.15
Lake water	10	10.33	103.30	2.03	101.46	10.21	102.10	3.14	101.81
	50	52.11	104.22	3.92	102.83	50.16	100.32	1.43	50.20
	100	105.33	105.33	2.05	103.27	101.92	101.92	2.15	100.65
River water	10	9.96	99.60	3.39	9.98	10.42	104.20	3.24	103.22
	50	52.01	104.02	1.99	100.62	51.09	102.18	1.30	101.53
	100	98.74	98.74	0.92	99.2	105.04	105.04	1.41	102.30

## Data Availability

The original contributions presented in this study are included in the article/Supplementary Material. Further inquiries can be directed to the corresponding author.

## Supplementary Materials

The following supporting information can be downloaded at: https://www.mdpi.com/article/10.3390/ma18122828/s1, Figure S1: Cyclic voltammetry of GCE (electrolyte: 0.1 M KCl and 5 mM [Fe(CN)_6_]^3−/4−^ mixed solution, deposition potential: −0.1~0.5 V); Figure S2: EDX mapping and EDX elemental mappings of Ti_3_AlC_2_ MXA (a–e); EDX mapping and EDX elemental mappings of Ti_3_C_2_T_x_ MXene (f–j); elemental distribution of C, Ti, O and Al, the illustration in figure (a) and (f) is SEM images of Ti_3_AlC_2_ MXA and Ti_3_C_2_T_x_ MXene; Figure S3: TEM and HRTEM image of Ti_3_AlC_2_ MAX (a,b), DL-Ti_3_C_2_T_x_ MXene (c,d), Bi/DL-Ti_3_C_2_T_x_ (e,f), the illustration is EDP of DL-Ti_3_C_2_T_x_ MXene (d), Bi/DL-Ti_3_C_2_T_x_ (f); Figure S4: XRD patterns of DL-Ti_3_C_2_T_x_ MXene and Bi/DL-Ti_3_C_2_T_x_; Figure S5: XPS image of Ti_3_AlC_2_ MAX; Figure S6: (a) Cyclic voltammetry and (b) Nyquist plots of the EIS for GCE, DL-Ti_3_C_2_T_x_/GCE and Bi/DL-Ti_3_C_2_T_x_/GCE in 5.0 mM K_3_[Fe(CN)_6_]^3−/4−^ containing 0.1 M KCl solution (the illustration is equivalent circuit); Figure S7: SWASV curves of Bi/DL-Ti_3_C_2_T_x_/GCE in 0.2 M ABS (pH = 4.5) containing 100 μg/L Cd (II) (a), 100 μg/L Pb(II) (b) and the mixture of 100 μg/L Cd (II), 100 μg/L Pb(II), 300 μg/L Bi(III (c), the four types of electrodes include GCE, Bi/GCE, DL-Ti_3_C_2_T_x_ /GCE and Bi/DL-Ti_3_C_2_T_x_ /GCE in 0.2 M ABS (pH=4.5) containing the mixture of 100 μg/L Cd (II) (a),100 μg/L Pb(II), 300 μg/L Bi(III); Figure S8: Effects of (a,b) Volume of DL-Ti_3_C_2_T_x_ MXene on GCE, (c,d) pH value on the striping peak current. (Electrolyte: 0.2 M ABS buffer; pH = 4.5; c[Bi(III)]: 300 μg/L; c[Pb(II)]: 100 μg/L; c[Cd(II)]: 100 μg/L; interference ions concentration 1000 μg/L; deposition time: 270 s; deposition potential: −1.20 V); Figure S9: Effects of (a,b) heavy metal ion concentration and Bi(III) concentration ratio, (c,d) Deposition time and (e,f) Deposition Potential. (Electrolyte: 0.2 M ABS buffer; pH = 4.5; c[Bi(III)]: 300 μg/L; c[Pb(II)]: 100 μg/L; c[Cd(II)]: 100 μg/L; interference ions concentration 1000 μg/L; deposition time: 270 s; deposition potential: −1.20 V); Figure S10: Stability Study: Bi/DL-Ti_3_C_2_T_x_/GCE sensor reproducibility test of five times in 0.2 M ABS (pH = 4.5) containing 100 μg/L Cd(II) and 100 μg/L Pb (II).

## References

[1] Ahmad T., Khan S., Rasheed T., Ullah N. (2022). Graphitic carbon nitride nanosheets as promising candidates for the detection of hazardous contaminants of environmental and biological concern in aqueous matrices. Microchim. Acta.

[2] Liu Y., Guan L., Tu Y., Ruan Z., Chen J., Xu Z., Wang R., Liu H., Liu Z. (2025). Development of high-performance MoS_2_ with nanofoam architecture for gaseous elemental mercury sequestration: The key role of edge sulfur vacancy. Chem. Eng. J..

[3] Liu J., Kang H., Tao W., Li H., He D., Ma L., Li X. (2023). A spatial distribution–Principal component analysis (SD-PCA) model to assess pollution of heavy metals in soil. Sci. Total Environ..

[4] He X., Deng F., Shen T., Yang L., Chen D., Lou J., Luo X., Min X., Wang F. (2019). Exceptional adsorption of arsenic by zirconium metal-organic frameworks: Engineering exploration and mechanism insight. J. Colloid Interface Sci..

[5] Wang C., Yang Z., Zhong C., Ji J. (2016). Temporal–spatial variation and source apportionment of soil heavy metals in the representative river–alluviation depositional system. Environ. Pollut..

[6] Huang Z., Chen J., Luo Z., Wang X., Duan Y. (2019). Label-free and enzyme-free colorimetric detection of Pb^2+^ based on RNA cleavage and annealing-accelerated hybridization chain reaction. Anal. Chem..

[7] Zhou X., Pu H., Sun D.W. (2021). DNA functionalized metal and metal oxide nanoparticles: Principles and recent advances in food safety detection. Crit. Rev. Food Sci..

[8] Shi J., Zhao D., Ren F., Huang L. (2023). Spatiotemporal variation of soil heavy metals in China: The pollution status and risk assessment. Sci. Total Environ..

[9] Saleh T.A., Mustaqeem M., Khaled M. (2022). Water treatment technologies in removing heavy metal ions from wastewater: A review. Environ. Nanotechnol. Monit. Manag..

[10] Yang X., Cheng B., Gao Y., Zhang H., Liu L. (2022). Heavy metal contamination assessment and probabilistic health risks in soil and maize near coal mines. Front. Public Health.

[11] Zhang Y., Song B., Zhou Z. (2023). Pollution assessment and source apportionment of heavy metals in soil from lead–Zinc mining areas of south China. J. Environ. Chem. Eng..

[12] Douvris C., Vaughan T., Bussan D., Bartzas G., Thomas R. (2023). How ICP-OES changed the face of trace element analysis: Review of the global application landscape. Sci. Total Environ..

[13] Chen W.T., Jiang S.J., Sahayam A.C. (2018). Speciation analysis of thallium in tobaccos using liquid chromatography inductively coupled plasma mass spectrometry. Microchem. J..

[14] Xing G., Sardar M.R., Lin B., Lin J.M. (2019). Analysis of trace metals in water samples using NOBIAS chelate resins by HPLC and ICP-MS. Talanta.

[15] Smirnova S.V., Samarina T.O., Ilin D.V., Pletnev I.V. (2018). Multielement determination of trace heavy metals in water by microwave-induced plasma atomic emission spectrometry after extraction in unconventional single-salt aqueous biphasic system. Anal. Chem..

[16] Shirani M., Habibollahi S., Akbari A. (2019). Centrifuge-less deep eutectic solvent based magnetic nanofluid-linked air-agitated liquid–liquid microextraction coupled with electrothermal atomic absorption spectrometry for simultaneous determination of cadmium, lead, copper, and arsenic in food samples and non-alcoholic beverages. Food Chem..

[17] Qian J., Gao X., Pan B. (2020). Nanoconfinement-mediated water treatment: From fundamental to application. Environ. Sci. Technol..

[18] Han Q., Yang X., Huo Y., Lu J., Liu Y. (2023). Determination of ultra-trace amounts of copper in environmental water samples by dispersive liquid-liquid microextraction combined with graphite furnace atomic absorption spectrometry. Separations.

[19] Liu Y., Qiu R., Zhang Z., Chen D., Gao Y., Liu Z., Wang C. (2022). Label-free electrochemical biosensor based on GR5 DNAzyme/Ti_3_C_2_T_x_ Mxenes for Pb^2+^ detection. J. Electroanal. Chem..

[20] Sulthana S.F., Iqbal U.M., Suseela S.B., Anbazhagan R., Chinthaginjala R., Chitathuru D., Kim T.H. (2024). Electrochemical sensors for heavy metal ion detection in aqueous medium: A systematic review. ACS Omega.

[21] Ariño C., Banks C.E., Bobrowski A., Crapnell R.D., Economou A., Królicka A., Wang J. (2022). Electrochemical stripping analysis. Nat. Rev. Methods Primes.

[22] Oularbi L., Turmine M., El Rhazi M. (2019). Preparation of novel nanocomposite consisting of bismuth particles, polypyrrole and multi-walled carbon nanotubes for simultaneous voltammetric determination of cadmium(II) and lead(II). Synth. Met..

[23] Yu L., Sun L., Zhang Q., Zhou Y., Zhang J., Yang B., Xu Q. (2022). Nanomaterials-based ion-imprinted electrochemical sensors for heavy metal ions detection: A review. Biosensors.

[24] Promsuwan K., Sanguarnsak C., Samoson K., Saichanapan J., Soleh A., Saisahas K., Limbut W. (2024). Single-drop electrodeposition of nanoneedle-like bismuth on disposable graphene electrode for on-site electrochemical detection of cadmium and lead. Talanta.

[25] Huang H., Wang J., Zheng Y., Bai W., Ma Y., Zhao X. (2024). A screen-printed carbon electrode modified with bismuth nanoparticles and conjugated mesoporous polymer for simultaneous determination of Pb (II) and Cd (II) in seafood samples. J. Food Compos. Anal..

[26] Wen L., Dong J., Yang H., Zhao J., Hu Z., Han H., Huo D. (2022). A novel electrochemical sensor for simultaneous detection of Cd^2+^ and Pb^2+^ by MXene aerogel-CuO/carbon cloth flexible electrode based on oxygen vacancy and bismuth film. Sci. Total Environ..

[27] Wu J., Wang Y., Zhang Y., Meng H., Xu Y., Han Y., Zhang X. (2020). Highly safe and ionothermal synthesis of Ti_3_C_2_ MXene with expanded interlayer spacing for enhanced lithium storage. J. Energy Chem..

[28] Gogotsi Y., Anasori B. (2019). The rise of MXenes. ACS Nano.

[29] Ho D.H., Choi Y.Y., Jo S.B., Myoung J.M., Cho J.H. (2021). Sensing with MXenes: Progress and prospects. Adv. Mater..

[30] Barmann P., Nolle R., Siozios V., Ruttert M., Guillon O., Winter M., Placke T. (2021). Solvent co-intercalation into few-layered Ti_3_C_2_T_x_ MXenes in lithium ion batteries induced by acidic or basic post-treatment. ACS Nano.

[31] Lu M., Li H., Han W., Wang Y., Shi W., Wang J., Zheng W. (2019). Integrated MXene & CoFe_2_O_4_ electrodes with multi-level interfacial architectures for synergistic lithium-ion storage. Nanoscale.

[32] Miao J., Zhu Q., Li K., Zhang P., Zhao Q., Xu B. (2021). Self-propagating fabrication of 3D porous MXene-rGO film electrode for high-performance supercapacitors. J. Energy Chem..

[33] Rasheed P.A., Pandey R.P., Gomez T., Naguib M., Mahmoud K.A. (2020). Large interlayer spacing Nb_4_C_3_T_x_ (MXene) promotes the ultrasensitive electrochemical detection of Pb^2+^ on glassy carbon electrodes. RSC Adv..

[34] Zukauskas S., Rucinskiene A., Ramanavicius S., Popov A., Niaura G., Baginskiy I., Ramanavicius A. (2024). Electrochemical real-time sensor for the detection of Pb(II) ions based on Ti_3_C_2_T_x_ MXene. Sci. Total Environ..

[35] Hou W., Sun Y., Zhang Y., Wang T., Wu L., Du Y., Zhong W. (2021). Mixed-dimensional heterostructure of few-layer MXene based vertical aligned MoS_2_ nanosheets for enhanced supercapacitor performance. J. Alloys Compd..

[36] Kumar S., Lei Y., Alshareef N.H., Quevedo-Lopez M.A., Salama K.N. (2018). Biofunctionalized two-dimensional Ti_3_C_2_ MXenes for ultrasensitive detection of cancer biomarker. Biosens. Bioelectron..

[37] Sun N., Zhu Q., Anasori B., Zhang P., Liu H., Gogotsi Y., Xu B. (2019). MXene-bonded flexible hard carbon film as anode for stable Na/K-ion storage. Adv. Funct. Mater..

[38] Guo X., Ding Y., Kuang D., Wu Z., Sun X., Du B., He Y. (2021). Enhanced ammonia sensing performance based on MXene-Ti_3_C_2_T_x_ multilayer nanoflakes functionalized by tungsten trioxide nanoparticles. J. Colloid Interface Sci..

[39] Anasori B., Lukatskaya M.R., Gogotsi Y. (2017). 2D metal carbides and nitrides (MXenes) for energy storage. Nat. Rev. Mater..

[40] Hong L.F., Guo R.T., Yuan Y., Ji X.Y., Li Z.S., Lin Z.D., Pan W.G. (2020). Recent progress of two-dimensional MXenes in photocatalytic applications: A review. Mater. Today Energy.

[41] Zhang H., Abe I., Oyumi T., Ishii R., Hara K., Izumi Y. (2024). Photocatalytic CO_2_ Reduction Using Ti_3_C_2_X_y_ (X = Oxo, OH, F, or Cl) MXene–ZrO_2_: Structure, Electron Transmission, and the Stability. Langmuir.

[42] Li T., Yao L., Liu Q., Gu J., Luo R., Li J., Zhang D. (2018). Fluorine-free synthesis of high-purity Ti_3_C_2_T_x_ (T = OH, O) via alkali treatment. Angew. Chem. Int. Ed..

[43] Rostami M., Badiei A., Ziarani G.M. (2024). A review of recent progress in the synthesis of 2D Ti_3_C_2_T_x_ MXenes and their multifunctional applications. Inorg. Chem. Commun..

[44] Shen L., Zhao W., Wang K., Xu J. (2021). GO-Ti_3_C_2_ two-dimensional heterojunction nanomaterial for anticorrosion enhancement of epoxy zinc-rich coatings. J. Hazard. Mater..

[45] Chen S., Wang Y., Zhang B., Li M., Zhang J., Hu Q., Liu H. (2024). Electrochemical Detection of Cd^2+^ and Pb^2+^ in Wastewater by Amino C-dot-MOF/GCE. ECS. J. Solid State Sci. Technol..

[46] Chen X., Zhao J.X., Wang J.W., Liu Y., Wang L.C., Weerasooriya R., Wu Y.C. (2021). Doping ZIF-67 with transition metals results in bimetallic centers for electrochemical detection of Hg(II). Electrochim. Acta.

[47] Wang W., Wu S. (2017). A new ternary composite based on carbon nanotubes/polyindole/graphene with preeminent electrocapacitive performance for supercapacitors. Appl. Surf. Sci..

[48] Tahaei R., Shayani-Jam H., Yaftian M.R. (2021). Voltammetric determination of trace copper(II), cadmium(II), and lead(II) using a Schiff base modified glassy carbon working electrode. Monatsh. Chem..

[49] Hui X., Sharifuzzaman M., Sharma S., Xuan X., Zhang S., Ko S.G., Park J.Y. (2020). High-performance flexible electrochemical heavy metal sensor based on layer-by-layer assembly of Ti_3_C_2_T_x_/MWNTs nanocomposites for noninvasive detection of copper and zinc ions in human biofluids. ACS Appl. Mater. Interfaces.

[50] Sun Y.F., Li P.H., Yang M., Huang X.J. (2019). Highly sensitive electrochemical detection of Pb(II) based on excellent adsorption and surface Ni(II)/Ni(III) cycle of porous flower-like NiO/rGO nanocomposite. Sens. Actuators B-Chem..

[51] Rosolina S.M., Chambers J.Q., Lee C.W., Xue Z.L. (2015). Direct determination of cadmium and lead in pharmaceutical ingredients using anodic stripping voltammetry in aqueous and DMSO/water solutions. Anal. Chim. Acta.

[52] Yang D., Wang L., Chen Z., Megharaj M., Naidu R. (2014). Voltammetric determination of lead(II) and cadmium(II) using a bismuth film electrode modified with mesoporous silica nanoparticles. Electrochim. Acta.

[53] Cerovac S., Guzsvány V., Kónya Z., Ashrafi A.M., Švancara I., Rončević S., Vytřas K. (2015). Trace level voltammetric determination of lead and cadmium in sediment pore water by a bismuth-oxychloride particle-multiwalled carbon nanotube composite modified glassy carbon electrode. Talanta.

[54] He Y., Wang Z., Ma L., Zhou L., Jiang Y., Gao J. (2020). Synthesis of bismuth nanoparticle-loaded cobalt ferrite for electrochemical detection of heavy metal ions. RSC Adv..

[55] Zhu X., Liu B., Hou H., Huang Z., Zeinu K.M., Huang L., Yang J. (2017). Alkaline intercalation of Ti_3_C_2_ MXene for simultaneous electrochemical detection of Cd(II), Pb(II), Cu(II) and Hg(II). Electrochim. Acta.

