# Supplementary material for: Simultaneously Selective Detection of Trace Lead and Cadmium Ions by Bi-Modified Delaminated Ti3C2Tx/GCE Sensor: Optimization, Performance and Mechanism Insights
Source: Materials (Basel). 2025 Jun 16;18(12):2828. doi: 10.3390/ma18122828 (PMC12194994; doi:10.3390/ma18122828)
Supplement: Supplementary file 1 [file materials-18-02828-s001.zip › materials-3609933-supplementary.pdf]

Supplemental Material

Simultaneously Selective Detection of Trace Lead and Cadmium Ions by Bi-Modified Delaminated $Ti_3C_2T_x$/GCE Sensor: Optimization, Performance and Mechanism Insights

Ruhua Peng [1,†], Kai Tao [1,†], Baixiong Liu [1], Jiayu Chen [1], Yunhang Zhang [1], Yuxiang Tan [1], Fuqiang Zuo [2], Caihua Song [2] and Xingyu He [1,2,*]

[1] School of Materials Science and Engineering, Jiangxi University of Science and Technology, Ganzhou 341000, China; 15207080615@163.com (R.P.); 15868100589@163.com (K.T.); liu_micro@126.com (B.L.); 13879375814@163.com (J.C.); 19532332665@163.com (Y.Z.); 19179998791@163.com (Y.T.)

[2] Sanchuan Wisdom Technology Co., Ltd., Yingtan 335000, China; a19532332665@outlook.com (F.Z.); tk15868100589@126.com (C.S.)

* Correspondence: hxyjxust@126.com

† The authors contributed equally to this work.

Text S1. The Shanghai Chenhua CHI 660E electrochemical workstation was purchased from Shanghai Chenhua Instrument Co., Ltd.(CHI 660E, Shanghai, China), and the constant temperature ultrasonic cleaner KQ-100DB was purchased from Kunshan Ultrasonic Instrument Co., Ltd.(Kunshan, China). The three-electrode system includes a working electrode: glassy carbon electrode (GCE, d=3 mm), and a reference electrode: 851 type Ag/AgCl (3M KCl); Counter electrode: Platinum wire electrode (diameter 1 mm × 5 mm), the above electrodes were all purchased from Shanghai Yueci Electronic Technology Co., Ltd.(Shanghai, China).

Titanium aluminum carbide (Ti_3AlC_2), lithium fluoride (LiF), cadmium nitrate ($Cd(NO_3)_2\cdot 4H_2O$), bismuth nitrate ($Bi(NO_3)_3\cdot 5H_2O$), lead nitrate ($Pb(NO_3)_2$) are sourced from Tianjin Komio Chemical Reagent Co.(Tianjin, China); Ltd. Potassium ferrocyanide ($K_3[Fe(CN)_6]$), potassium ferrocyanide ($K_4[Fe(CN)_6]\cdot 3H_2O$) are

purchased from Shanghai McLean Biochemical Technology Co., Ltd.(Shanghai, China); Potassium chloride (KCl), concentrated hydrochloric acid (HCl), nitric acid (HNO_3), sodium acetate (CH_3COONa), glacial acetic acid (CH_3COOH), ethanol (C_2H_5OH) were purchased from Xilong Science Co., Ltd.(Shantou, China); and aluminum oxide powder (Al_2O_3, 0.3 μm, 0.05 μm) was purchased from Shanghai Yueci Electronic Technology Co., Ltd.(Shanghai, China); All reagents are analytical grade and directly used without further purification. All experiments were conducted using ultrapure water (H_2O, self-made in the laboratory) with a resistivity of 18.2 MΩ cm^{-1}.

Text S2. In three electrode system, the treated glassy carbon electrode (GCE) is used as the working electrode, the platinum wire electrode is used as the counter electrode, and the Ag/AgCl (3M KCl) electrode is used as the reference electrode. In a mixed solution of 0.1 M KCl and 5 mM $[Fe(CN)_6]^{3-/4-}$, cyclic voltammetry is performed within the voltage range of -0.1~0.5 V. The DL-$Ti_3C_2T_x$/GCE was used as the working electrode, Ag/AgCl (3M KCl) as the reference electrode, platinum wire electrode as the counter electrode, and the electrolyte was 0.2 mol/L Acetate Buffer Saline (ABS) (pH=4.5) buffer containing Bi^{3+}. In the cyclic voltammetry (CV) experiment, a mixture of 0.1M KCl and 0.5 mM $[Fe(CN)_6]^{3-/4-}$ electrolyte was used, with a voltage range of -0.1~ 0.5 V.

Text S3. In order to comprehensively understand the structural and morphological characteristics of the prepared nanomaterials, Scanning electron microscopy (SEM&EDS; Carl Zeiss, Germany) was used to investigate the microstructure and elemental distribution of the sample. The microstructure of the sample was analyzed

using transmission electron microscopy (TEM; Tecnai G220, USA). By X-ray diffraction (XRD; SHIMADZU XRD-7000, Japan), the phase composition and crystal structure information of the sample were analyzed in the range of 2-70° (2θ) under the following measurement conditions: λ=1.54 Å, step size of 0.02°, and scanning speed of 2°/min. The surface elemental composition and chemical valence states of the sample were determined using X-ray photoelectron spectroscopy (XPS; Thermo Scientific K-Alpha, USA) at an Al Kα ray energy of 1486.6 eV.

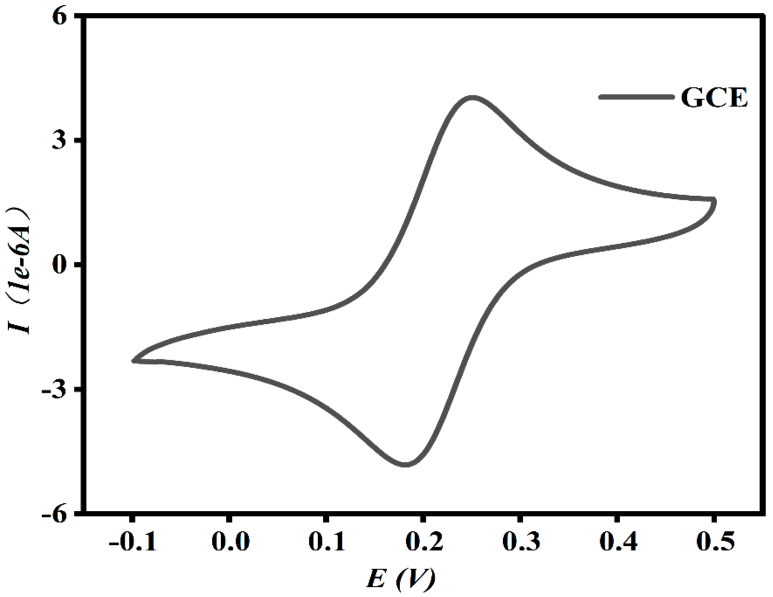

Figure S1 Cyclic voltammetry of GCE (electrolyte: 0.1 M KCl and 5 mM $[Fe(CN)_6]^{3-/4-}$ mixed solution, deposition potential: -0.1~0.5 V)

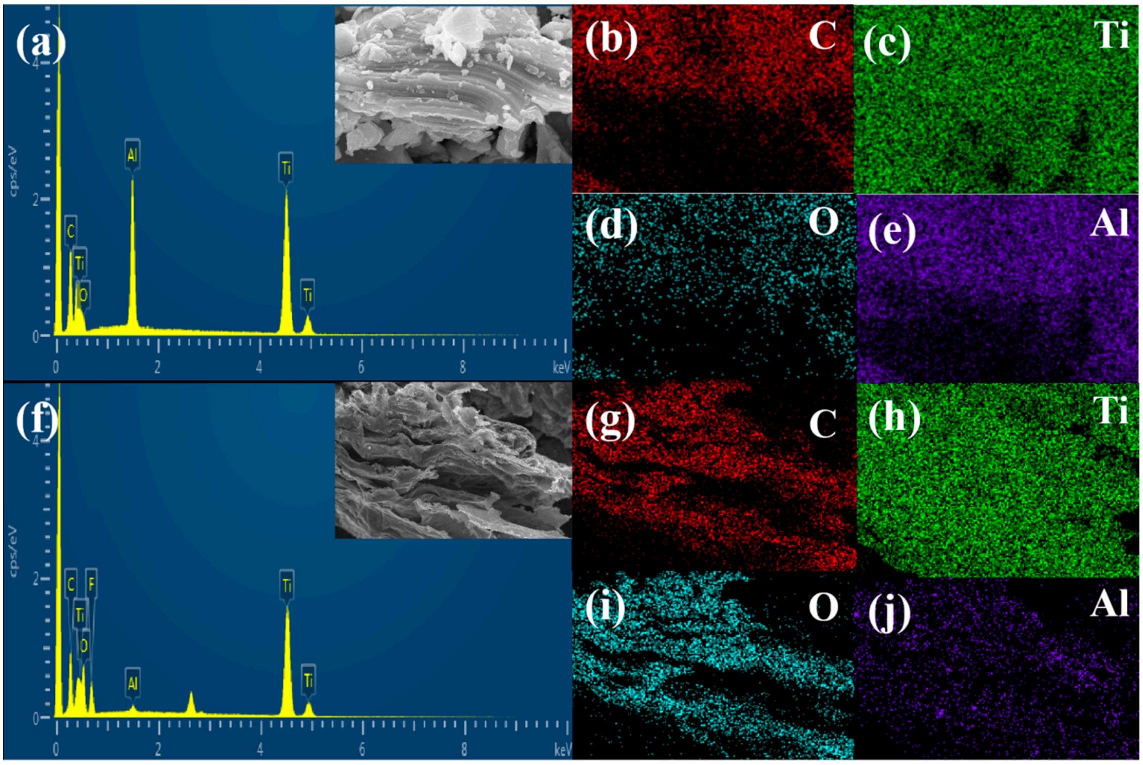

Figure S2 EDX mapping and EDX elemental mappings of Ti_3AlC_2 MXA (a-e); EDX mapping and EDX elemental mappings of $Ti_3C_2T_x$ MXene (f-j); elemental distribution of C, Ti, O and Al, the illustration in figure (a) and (f) is SEM images of Ti_3AlC_2 MXA and $Ti_3C_2T_x$ MXene.

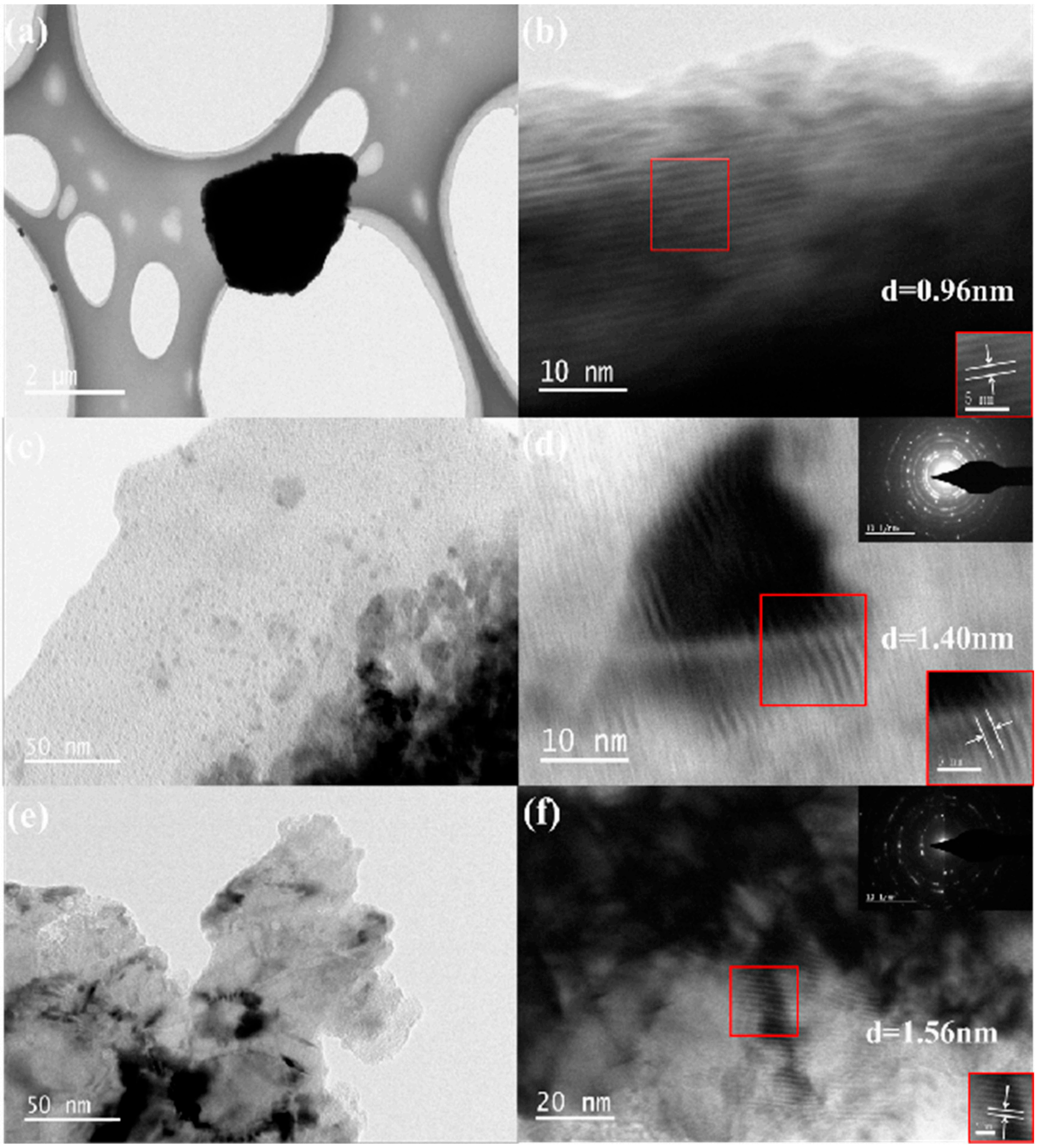

Figure S3 TEM and HRTEM image of Ti_3AlC_2 MAX (a-b), DL-$Ti_3C_2T_x$ MXene (c-d), Bi/DL-$Ti_3C_2T_x$ (e-f), the illustration is EDP of DL-$Ti_3C_2T_x$ MXene (d), Bi/DL-$Ti_3C_2T_x$ (f).

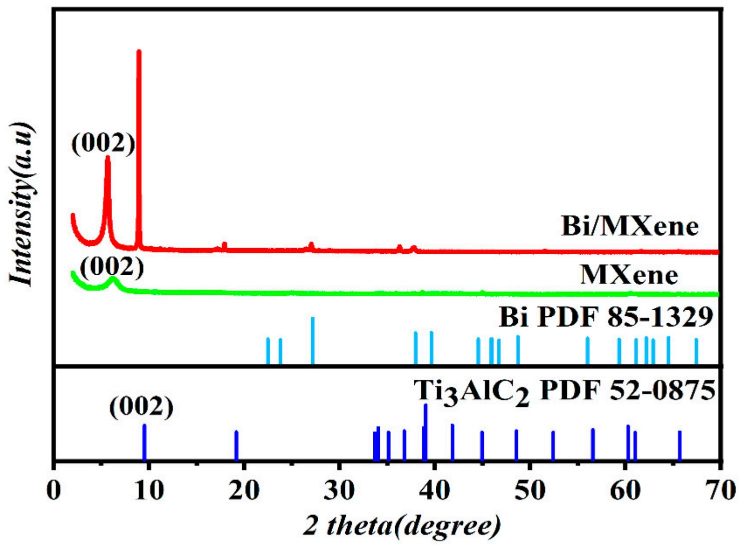

Figure S4 XRD patterns of DL-$Ti_3C_2T_x$ MXene and Bi/DL-$Ti_3C_2T_x$.

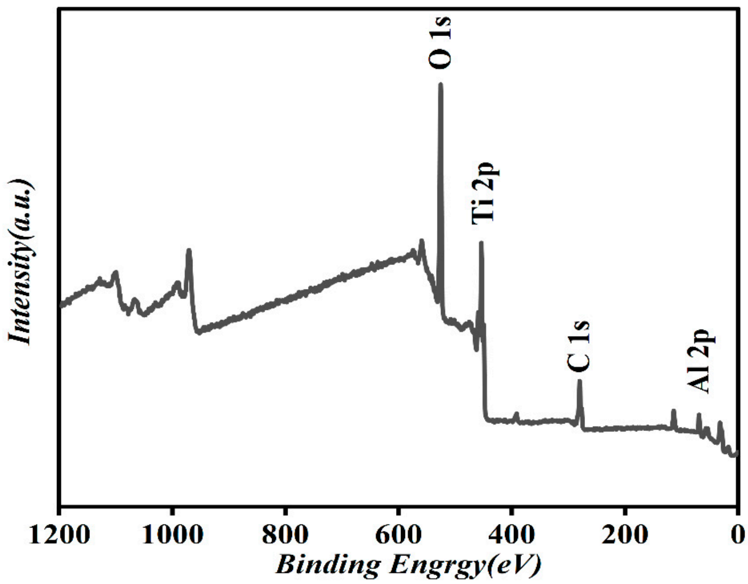

Figure S5 XPS image of Ti_3AlC_2 MAX.

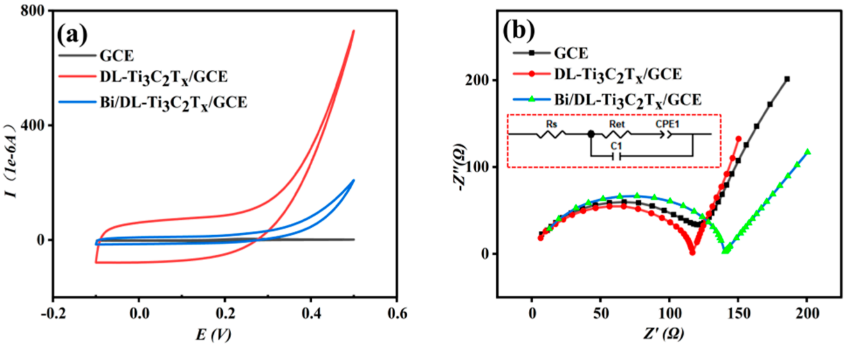

Figure S6 (a) Cyclic voltammetry and (b) Nyquist plots of the EIS for GCE, DL-$Ti_3C_2T_x$/GCE and Bi/DL-$Ti_3C_2T_x$/GCE in 5.0 mM $K_3[Fe(CN)_6]^{3-/4-}$ containing 0.1 M KCl solution (the illustration is equivalent circuit).

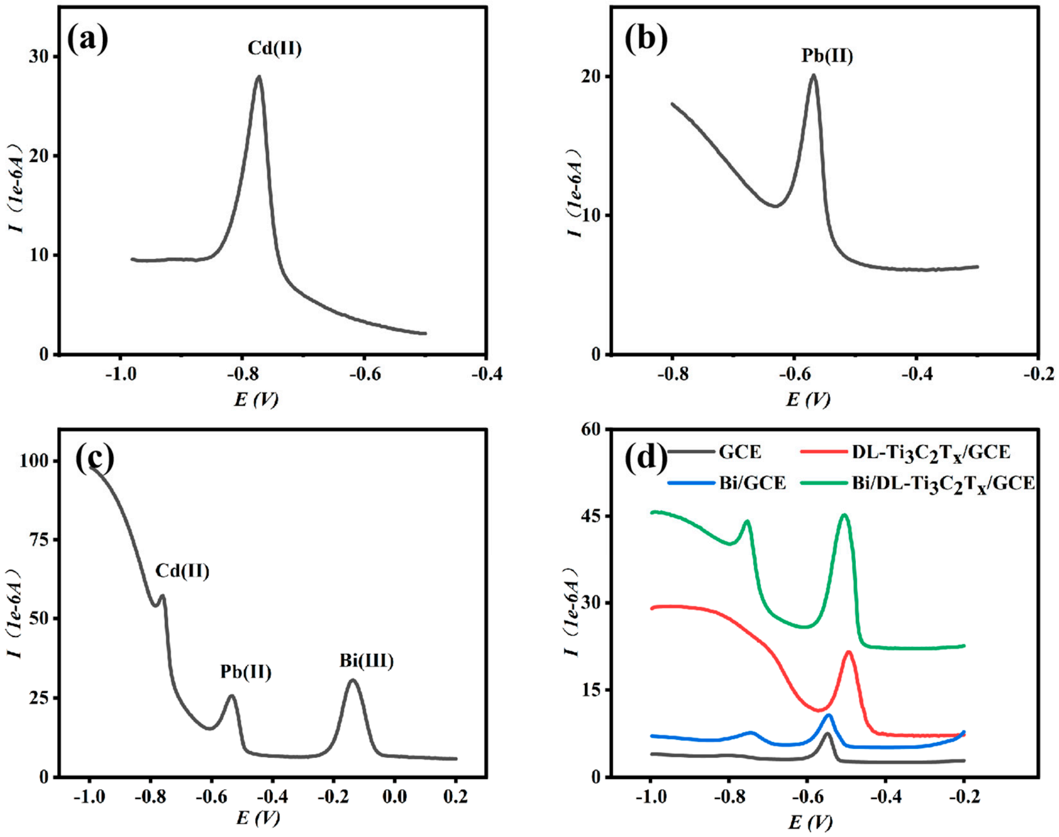

Figure S7 SWASV curves of Bi/DL-$Ti_3C_2T_x$/GCE in 0.2 M ABS (pH=4.5) containing 100 µg/L Cd (II) (a), 100 µg/L Pb(II) (b) and the mixture of 100 µg/L Cd (II),100 µg/L Pb(II) ,300 µg/L Bi(III) (c), the four types of electrodes include GCE, Bi/GCE, DL-$Ti_3C_2T_x$/GCE and Bi/DL-$Ti_3C_2T_x$/GCE in 0.2 M ABS (pH=4.5) containing the mixture of 100 µg/L Cd (II) (a),100 µg/L Pb(II), 300 µg/L Bi(III).

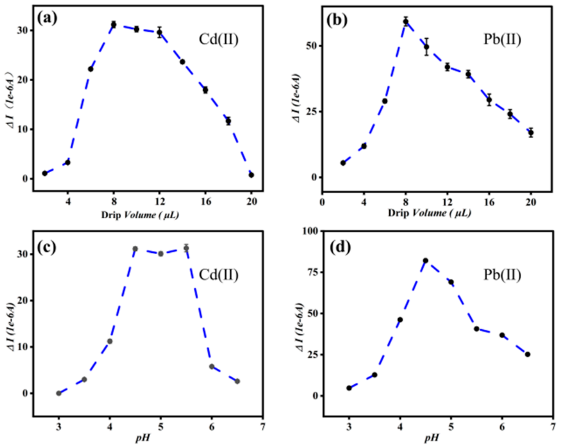

Figure S8 Effects of (a-b) Volume of DL-$Ti_3C_2T_x$ MXene on GCE, (c-d) pH value on the striping peak current. (Electrolyte: 0.2 M ABS buffer; pH=4.5; c[Bi(III)]: 300 μg/L; c[Pb(II)]: 100 μg/L; c[Cd(II)]: 100 μg/L; interference ions concentration 1000 μg/L; deposition time: 270 s; deposition potential: -1.20 V)

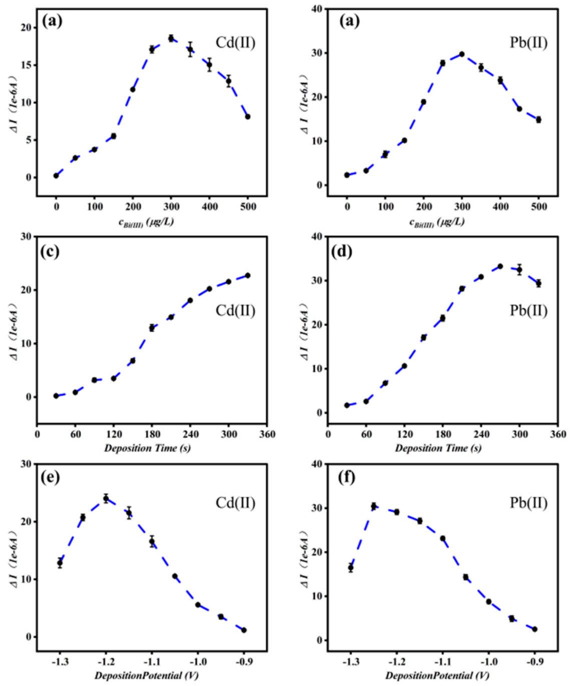

Figure S9 Effects of (a-b) heavy metal ion concentration and Bi(III) concentration ratio, (c-d) Deposition time and (e-f) Deposition Potential. (Electrolyte: 0.2 M ABS buffer; pH=4.5; c[Bi(III)]: 300 μg/L; c[Pb(II)]: 100 μg/L; c[Cd(II)]: 100 μg/L; interference ions concentration 1000 μg/L; deposition time: 270 s; deposition potential: - 1.20 V)

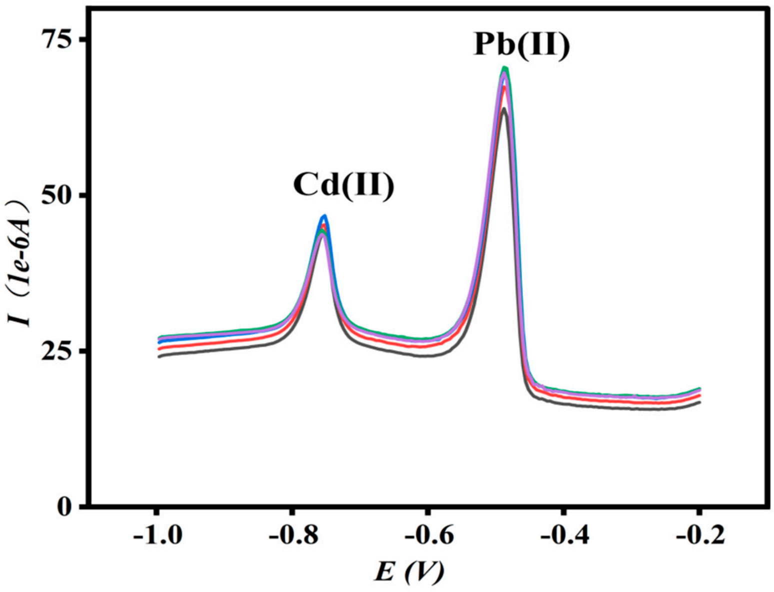

Figure S10 Stability Study: Bi/DL-$Ti_3C_2T_x$/GCE sensor reproducibility test of five times in 0.2 M ABS (pH=4.5) containing 100 μg/L Cd(II) and100 μg/L Pb (II).
